# Comparing gut resistome composition among patients with acute *Campylobacter* infections and healthy family members

**DOI:** 10.1038/s41598-021-01927-7

**Published:** 2021-11-16

**Authors:** Zoe A. Hansen, Wonhee Cha, Brian Nohomovich, Duane W. Newton, Paul Lephart, Hossein Salimnia, Walid Khalife, Ashley Shade, James T. Rudrik, Shannon D. Manning

**Affiliations:** 1 Departments of Microbiology and Molecular Genetics, Michigan State University, East Lansing, MI 48824 USA; 2 Plant, Soil and Microbial Sciences, Michigan State University, East Lansing, MI 48824 USA; 3 Ecology, Evolution, and Behavior Program, Michigan State University, East Lansing, MI 48824 USA; 4 University of Michigan, Ann Arbor, MI 48109 USA; 5 Wayne State University, Detroit, MI 48202 USA; 6 Sparrow Hospital, Lansing, MI 48912 USA; 7 Michigan Department of Health and Human Services, Bureau of Laboratories, Lansing, MI 48913 USA

**Keywords:** Microbiome, Gastroenteritis

## Abstract

*Campylobacter* commonly causes foodborne infections and antibiotic resistance is an imminent concern. It is not clear, however, if the human gut ‘resistome’ is affected by *Campylobacter* during infection. Application of shotgun metagenomics on stools from 26 cases with *Campylobacter* infections and 44 healthy family members (controls) identified 406 unique antibiotic resistance genes (ARGs) representing 153 genes/operons, 40 mechanisms, and 18 classes. Cases had greater ARG richness (p < 0.0001) and Shannon diversity (p < 0.0001) than controls with distinct compositions (p = 0.000999; PERMANOVA). Cases were defined by multidrug resistance genes and were dominated by Proteobacteria (40.8%), specifically those representing *Escherichia* (20.9%). Tetracycline resistance genes were most abundant in controls, which were dominated by Bacteroidetes (45.3%) and Firmicutes (44.4%). Hierarchical clustering of cases identified three clusters with distinct resistomes. Case clusters 1 and 3 differed from controls containing more urban and hospitalized patients. Relative to family members of the same household, ARG composition among matched cases was mostly distinct, though some familial controls had similar profiles that could be explained by a shorter time since exposure to the case. Together, these data indicate that *Campylobacter* infection is associated with an altered resistome composition and increased ARG diversity, raising concerns about the role of infection in the spread of resistance determinants.

## Introduction

Enteric pathogens are common causes of foodborne illness affecting 9.4 million individuals each year in the United States; 3.6 million of these enteric infections are caused by bacteria^1^. In 2018, the Centers for Disease Control and Prevention (CDC) reported that the incidence of foodborne infection was highest for *Campylobacter* and *Salmonella*, with the incidence of both pathogens increasing relative to the frequencies reported in 2015–2017^2^. The pathogenesis and virulence of these enteric pathogens have been well characterized and, more recently, several studies have examined how enteric pathogens influence the gut microbiota. For example, our prior 16S rRNA sequencing study showed that infection by one of four enteric pathogens resulted in decreased diversity of the gut microbiota, specifically resulting in an increase in the relative abundance of *Proteobacteria*^3^. Thus, further consideration and characterization of the ecological consequences of enteric infection in the human gut microbiome is needed.

In addition to their role in causing foodborne illness, *Campylobacter* spp., are progressively found to be drug-resistant, which has led to their classification as a serious public health threat by the CDC^4^. The increasing prevalence of *Campylobacter* spp. linked to human infections plus their enhanced ability to evade modern antibiotics substantiate the need to further understand their total impact on health. Generally, antibiotic resistance increasingly results in adverse health and economic outcomes due to the growing prevalence and emergence of drug-resistant infections^1,5^. Growing awareness of these burdens has led to a rise in the number of studies investigating the resistome, or the compilation of antimicrobial resistance genes (ARGs), within microbial communities^6^. Several studies have investigated resistomes across different environments including the guts of humans, cattle, poultry, and swine^7–9^. These environments do not exist in isolation; one study found similar genetic regions containing ARGs among environmental soil isolates and five relevant human pathogens^10^, while another identified ARGs that could cross habitat boundaries^11^. Many of these genes are co-localized with mobile genetic elements and other ARGs, suggesting significant potential for transmission of multiple resistance genes via horizontal gene transfer. This spread of antimicrobial resistance across environments illuminates our need to further clarify the ecological mechanisms facilitating such exchange.

As in other ecosystems, the human gut microbial community exhibits microbe-microbe and microbe-host interactions, temporal and spatial dynamics, and has varied community responses to disturbance or species invasion^12^. Multiple studies have explored the ability of the human gut microbiota to recover after a disturbance like antibiotic treatment^13,14^. One longitudinal study, for instance, investigated the effects of repeated antibiotic exposure in infants and found that antibiotic use contributed to a loss of species- and strain-level diversity^15^. Just as disturbance has the capacity to uproot stable communities, so, too, does microbial invasion. Microbial invasion ecology involves the introduction of a foreign microbe to a stable environment and follows a trajectory from establishment to growth and spread, leading to downstream ecological consequences^16^. Previous studies have examined the importance of microbial invasion in various environmental contexts such as soil, plant, and agricultural settings^17,18^. However, investigation of microbial invasion as it pertains to the human gut microbiome and resistome via infection has yet to be fully explored. Elucidating the impacts of ecological invasion on the composition and mobility of ARGs in the human gut is crucial to advancing our fight against the spread of drug resistance.

Given the health and economic burden of foodborne pathogens and the ubiquity of antimicrobial resistance across environments, further understanding the impacts of infection on ARGs and their dissemination is needed. This study therefore aims to understand enteric infection by a bacterial pathogen, *Campylobacter*, on the human gut resistome using shotgun metagenome analyses.

## Results

### Characteristics of the study population

Stools from 26 patients with acute campylobacteriosis (cases) were compared to stools from 44 related healthy family members (controls). Controls belonged to 16 different families with two to eight participating members. Although 10 cases and seven controls were not matched to a family, they were included in the comparative case versus control analyses. Among cases, 17 (65.4%) were female with 13 (50%) between 19 and 64 years of age, 8 (30.7%) between 0 and 9 years old, and 5 (19.2%) greater than 65 years (Table S1). Controls had a slightly different demographic distribution in which 18 (40.9%) were female; 17 (38.7%) were between 0 and 9 years old, 4 (9.1%) were 10–18 years of age, 21 (47.7%) were 19–64 years, and 2 (4.5%) were greater than 65 years old. No significant differences were observed in the age or sex distribution between groups. Although more controls resided in urban areas, the difference was not significant and is likely due to the recruitment of more than one control per case in most households. The majority of cases self-identified as Caucasian (n = 22; 88.0%) and reported abdominal pain (n = 21; 80.1%) and diarrhea (n = 24; 92.3%). Nausea (n = 9; 34.6%), vomiting (n = 6; 23.1%), and bloody stool (n = 10; 38.5%) were also reported, while 20 cases (76.0%) received outpatient care and six (23%) required hospitalization. Among the latter, four (66.7%) were hospitalized for two days, one for three days, and another for six. Three of the 26 cases (11.5%) and three of the 44 controls (6.8%) received antibiotics two weeks prior to sample collection.

### Number and diversity of ARGs vary depending on health status

**Figure 1:**
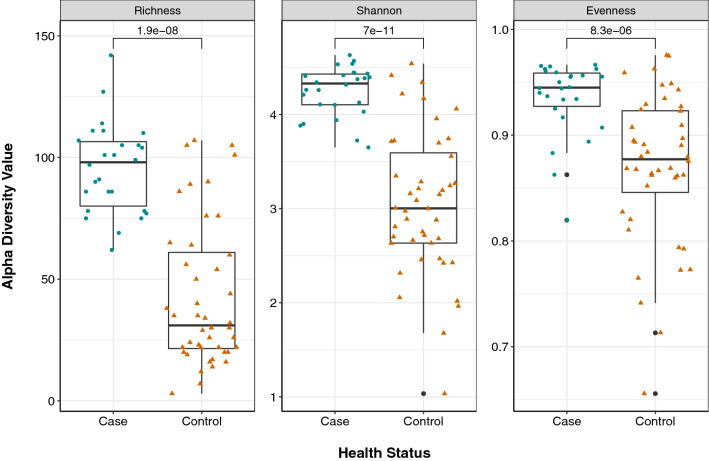
Resistomes in cases are more diverse than resistomes of controls. Three measures of alpha diversity (Richness, Shannon diversity, and Pielou’s Evenness) are shown stratified by health status. The median of each measure is indicated by the thick black bar in each box and the first and third quartiles are represented by the bottom and top of the box, respectively; points (circles and triangles) show variation within each sample type. Outlying points within each group are indicated by the black dots associated with each boxplot. P-values were calculated using the Wilcoxon rank-sum test and are shown above the comparison bar within each plot.

In total, 406 unique genes representing 153 ARG groups or operons for 18 antibiotic classes and 40 resistance mechanisms were detected. Three measures of alpha diversity (ARG richness, Shannon diversity, and evenness) differed significantly between groups (Fig. 1). The mean richness, or unique ARGs per sample, was greater in cases (S = 95.7; min = 62, max = 142) than controls (S = 42.8; min = 3, max = 107; p < 0.0001) as were the mean Shannon Diversity Index (cases = 4.25 vs. controls = 3.05; p < 0.0001) and resistome evenness (J’ = 0.935 (cases) vs. J’ = 0.869 (controls); p < 0.0001).

**Figure 2:**
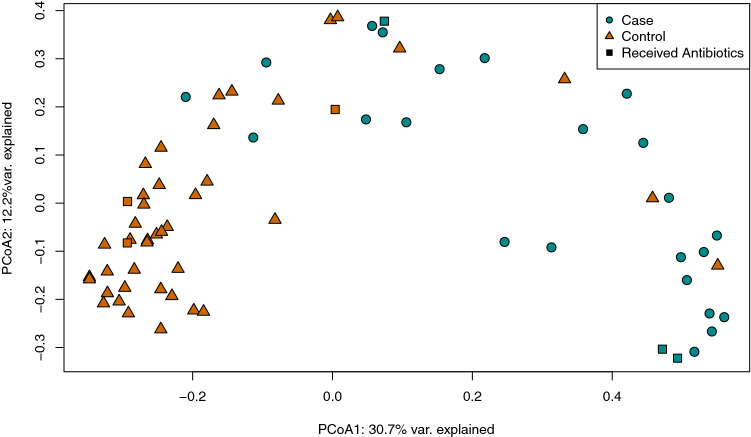
Resistomes of cases and controls are distinct. Principal Coordinates Analysis (PCoA) plot of case (cyan, circles) and control (orange, triangles) resistomes based on Bray–Curtis dissimilarity. The first and second coordinate are shown with their respective percentage of explained variance. Patients that self-reported use of antibiotics two weeks prior to sample collection are indicated by square data points.

Principal Coordinate Analysis (PCoA) using Bray–Curtis dissimilarity also revealed separation between case and control resistomes (Fig. 2). Indeed, health status, or identity as a case or control, had a significant effect on the centroid of each group as assessed using Permutational Analysis of Variance (PERMANOVA p = 0.000999; F = 14.083). The dispersion of points within each cluster evaluated using Permutational Analysis of Multivariate Dispersion, however, was not significantly different (PERMDISP p = 0.115; F = 2.6264), suggesting that the comparison between group centroids is valid. Participants reporting antibiotic use two weeks prior to sample collection did not cluster separately from other samples within each group.

### Specific ARGs define case and control samples

**Figure 3:**
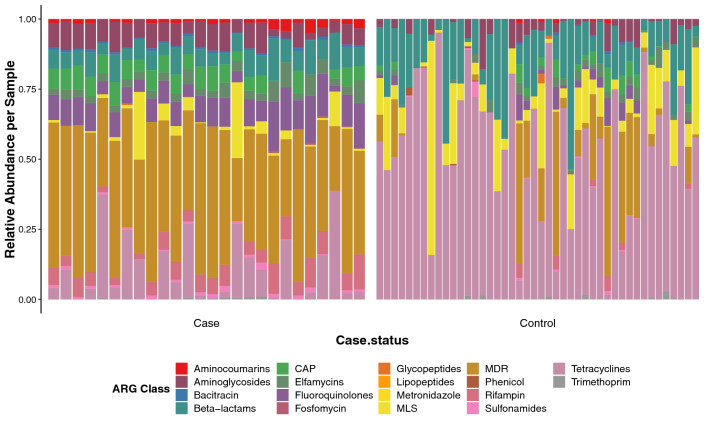
Relative abundance of ARGs differs in cases and controls. The relative abundance of ARGs assigned to 18 different antibiotic classes is shown with each column representing the resistome from one individual. Relative abundances were determined using raw ARG abundances that were normalized by the approximate number of genome equivalents in the sample as determined using MicrobeCensus. *CAP* cationic antimicrobial peptides, *MLS* Macrolide, Lincosamide, Streptogramin, *MDR* Multidrug resistance.

At the antibiotic class level, ARGs encoding for multidrug resistance (MDR), or resistance in three or more antibiotic classes, had the highest average relative abundance (42.6%) in cases followed by ARGs for tetracycline (11.0%) and fluoroquinolone (9.5%) (Fig. 3). For controls, tetracycline (54.4%) and beta-lactam (16.0%) ARGs were most common. At the group level (i.e., gene or operon), *tetQ* encoding tetracycline resistance was most abundant in both cases (8.0%) and controls (33.0%). In cases, the next highest groups were *mdtC* (5.9%) encoding a MDR efflux pump subunit in MdtBC and *rpoB* (5.4%)*,* the beta 30S RNA polymerase subunit gene important for rifampin, glycopeptide and lipopeptide resistance. Controls had a greater relative abundance of *tetW* (11.7%) encoding a ribosomal protection protein important for tetracycline resistance and the class A beta-lactamase *cfx* (11.1%). Among both sets of samples, the three respective predominant ARG groups represented ~ 60% of ARGs in controls compared to < 20% of the ARGs in case resistomes, further highlighting the increased resistome diversity within case communities.

**Figure 4:**
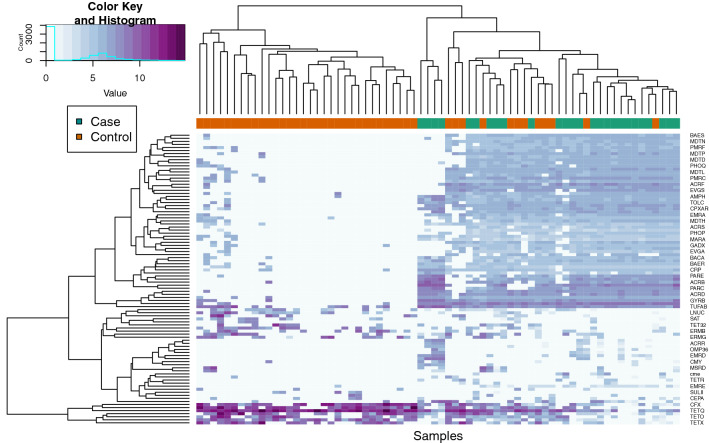
Hierarchical clustering illustrates group level ARG abundance differences between cases and controls. The columns represent the resistome communities per sample, which are ordered based on similarity in the top X-axis dendrogram that displays two resistome clusters. Case and control samples are indicated by the color bar below the dendrogram (cases = cyan; controls = orange). The Y-axis shows the hierarchical clustering of ARG groups as they appear in sample resistomes; ARG group names are indicated in small print on the right. Those ARG groups with a cumulative normalized abundance value < 5 across all samples were excluded from the analysis. Relative abundance is indicated by the color key; a value of 15 (deep purple) indicates that there are approximately 15 normalized copies of that ARG in a sample, while a value of 0 (light blue/white) indicates a very low or negligible abundance.

Normalizing by the number of genome equivalents per sample also detected differences in actual ARG abundance. Roughly 1,216 MDR genes were detected in cases versus 160 in controls. Cases also had more fluoroquinolone (n = 254) and aminoglycoside (n = 204) resistance genes than controls (n = 26, n = 31, respectively), while controls had more tetracycline ARGs (n = 270) than cases (n = 101). Moreover, clear differences in ARG abundance were observed across samples at the group level and hierarchical clustering revealed two distinct resistome clusters (Fig. 4). Of these clusters, one is comprised entirely of controls (n = 28) and the other contains samples from all 26 cases and 12 controls.

MaAsLin2^19^ was used to generate log-transformed linear models to identify differentially abundant ARGs among cases and controls. These models used health status as a fixed effect and residence type, age, and sex as random effects. At the class level, tetracycline, MLS, and beta-lactam ARGs were significantly associated with controls (adjusted p-values = 2.2E−11; 0.004; 0.021, respectively). In cases, the greatest association was observed for MDR (C = − 4.69; adjusted p-value = 4.01E−11) and fluoroquinolone resistance (C = − 4.37; adjusted p-value = 2.3E−12) relative to controls. At the gene level, increased abundance of MDR and fluoroquinolone resistance genes such as *cpxAR*, *mdtC*, *parE*, and *parC*, was observed among cases after adjusting for demographic variables (Table S2). Comparatively, tetracycline (*tetQ* and *tetW*) and beta-lactam resistance genes (*cfx* and *cbla*) were associated with controls. Similar ARG classes (Fig. S1) and ARGs (Fig. S2) were also found to differentiate case and control samples using Linear Discriminant Analysis (LDA) Effect Size (LEfSe)^20^.

### Taxonomic diversity differs between cases and controls

**Figure 5:**
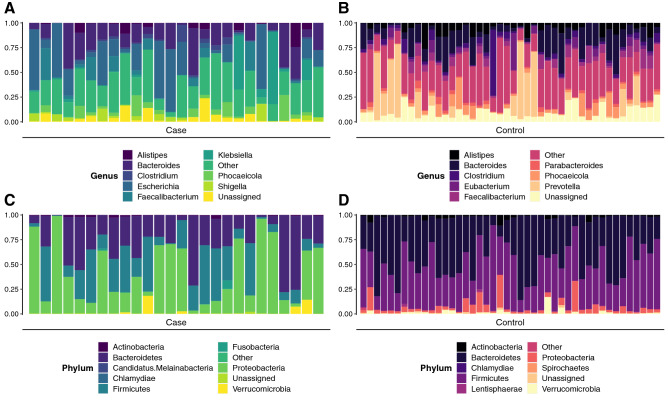
Taxonomic relative abundance notably differs between cases and controls. The relative abundance of bacterial genera and phyla in each sample are displayed as columns for cases (**A**, **C**) and controls (**B**, **D**). Similar to ARG relative abundance, taxonomic relative abundances were determined using raw abundances that had been normalized by the approximate number of genome equivalents in the sample as determined using MicrobeCensus. For the phylum and genus levels, the top-10 phyla and genera were chosen, respectively, based on the highest average relative abundance assigned to a specific phylum or genus among cases or controls (which were considered separately). The remaining read abundances for phyla or genera in samples were summed and are shown in the category “Other.” Note: plots for cases (**A**, **C**) and controls (**B**, **D**) contain the same respective color schemes but that these refer to genera (**A**, **B**) and phyla (**C**, **D**), respectively.

A total of 40,227 species were detected among the case and control samples including bacteria, archaea, fungi, and viruses. Mean taxonomic richness was significantly greater in controls (S = 6374; min = 1506, max = 15,548) compared to cases (S = 3605; min = 1,99, max = 11,612; p < 0.0001), a trend that was also observed for Shannon diversity (case = 3.36, control = 4.24; p = 0.00014) (Fig. S3). Expectedly, taxonomic composition was also distinct among cases and controls (Fig. 5). Cases were mostly comprised of *Proteobacteria* (average relative abundance = 40.8%) followed by *Bacteroidetes* (30.8%) represented primarily by the genera *Escherichia* (20.9%) and *Bacteroides* (18.1%), respectively. Conversely, controls were dominated by Bacteroidetes and Firmicutes with average relative abundances of 45.3% and 44.4%, respectively. The most highly represented genera in controls were *Bacteroides* (15.5%) and *Prevotella* (12.8%), both members of the *Bacteroidetes* phylum. Notably, a single control sample contained a high proportion of *Prevotella*, which accounted for 78% of its taxonomic abundance; with this outlying sample removed, the average relative abundance across controls for *Prevotella* was 11.2%.

The actual abundances of these bacterial groups also differed in taxonomic composition between cases and controls (Fig. S4). While cases had an average of 472 reads assigned to the genus *Escherichia*, controls had an average of just 37 *Escherichia* reads. Controls were dominated by *Prevotella* with an average of 1626 reads, though this high number was due to the outlier sample containing a high abundance of *Prevotella*. With this sample removed, the average number of *Prevotella* reads across controls was 411. Conversely, cases had an average of 75 *Prevotella* reads per sample. Among all cases, *Campylobacter* only comprised an average relative abundance of 0.28% at the time of sample collection. When considering actual abundance, cases had an average of only 4.0 *Campylobacter* reads per sample.

### Specific ARGs are not strongly associated with *Campylobacter* in the case samples

An analysis exploring correlations between the genus *Campylobacter* and ARG groups was pursued to investigate the potential role of the invading pathogens in shaping case resistomes. Spearman rank correlations between ARG and taxonomic abundances in cases were taken with a cutoff value of ρ ≥ 0.75. Although no significant correlations were observed between *Campylobacter* and other taxa or ARGs above this cutoff, statistically significant correlations with lower coefficients were detected. Namely, *Campylobacter* was positively correlated with *cme*, a gene encoding a class A beta-lactamase (coeff = 0.585; p = 0.00169), and *cmeR* that encodes a MDR efflux pump (coeff = 0.505; p = 0.00857). No other correlations with coefficients > 0.50 were observed between *Campylobacter* and ARGs in case samples.

Intriguingly, ARGs that best defined case samples were correlated with other taxa identified in case and control metagenomes (Table S3). For example, *mdtC* was highly abundant and positively correlated with *Shigella* (coeff = 0.886; p < 0.0001)*, Pseudoalteromonas* (coeff = 0.789; p < 0.0001)*, Rhodococcus* (coeff = 0.785; p < 0.0001)*,* and *Phytobacter* (coeff = 0.756; p < 0.0001). Although most of these genera were not overly abundant in cases, *Shigella* was among the top-10 most abundant genera. In addition, *cpxAR*, which encodes a regulatory system for a MDR efflux pump and was highly abundant in cases, was positively correlated with *Pseudoalteromonas* (coeff = 0.839; p < 0.0001)*, Phytobacter* (coeff = 0.793; p < 0.0001)*,* and *Siccibacter* (coeff = 0.784; p < 0.0001); however, none of these were among the top-100 most abundant genera.

### Hierarchical clustering detects three resistome clusters among cases

**Figure 6:**
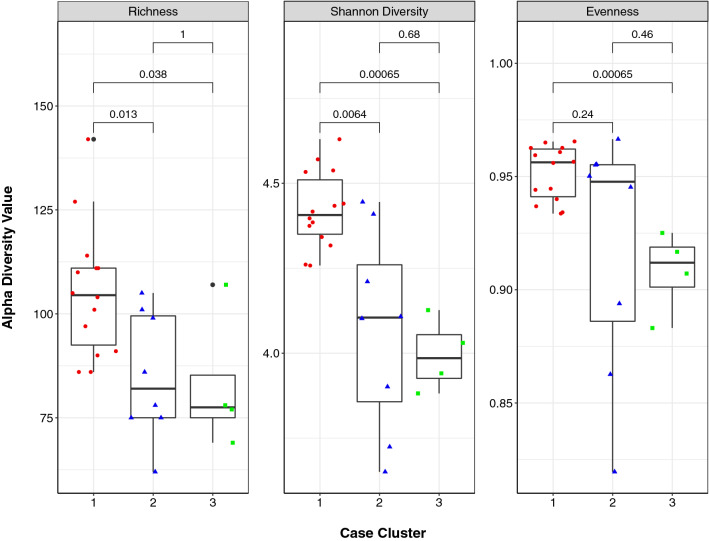
Case Cluster 1 resistomes are more diverse than resistomes of Clusters 2 and 3 combined**.** Boxplots displaying alpha diversity metrics for resistomes representing case Cluster 1 (red circles), Cluster 2 (blue triangles) and Cluster 3 (green squares) are shown. Resistome richness, diversity (Shannon), and evenness are indicated. The median of each measure is shown by the black bar within each box and the first and third quartiles are indicated by the bottom and top of the box, respectively; points show variation within sample types. P-values were calculated using the Wilcoxon rank-sum test and are shown above the comparison bar within each plot.

**Figure 7:**
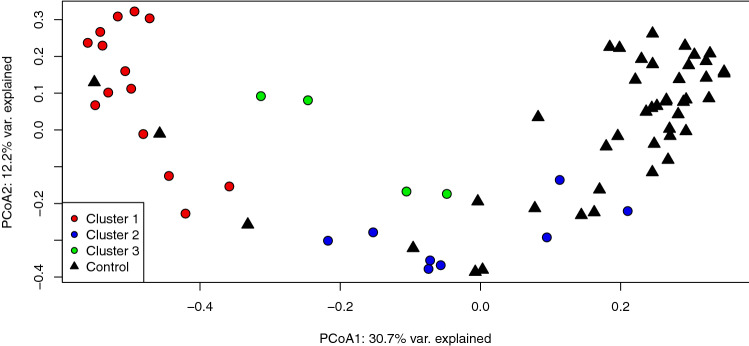
Case resistomes cluster separately and case Cluster 2 is more similar to control samples as shown in a Principal Coordinates Analysis (PCoA) plot of the three case clusters (red, blue, green; circles) compared to the control resistomes (black; triangles) based on Bray–Curtis dissimilarity at the gene level. The first and second coordinate are shown with their respective percentage of explained variance. Case Cluster 1 separates clearly from Clusters 2 and 3 along the first and second coordinate, while case Cluster 2 aligns with a handful of control samples.

Using Bray-Curtis dissimilarity, hierarchical clustering of case resistomes at the gene level identified three separate clusters among the 26 case samples (Fig. S5). The Cluster 1 cases had a significantly greater mean ARG richness (S = 105) than Cluster 2 (S = 85.1) and Cluster 3 (S = 82.8) cases (p = 0.01 and p = 0.04, respectively; Wilcoxon rank-sum test) (Fig. 6). Cluster 1 resistomes also had a significantly greater Shannon Diversity Index than Clusters 2 (p = 0.006) and 3 (p = 0.0007) and a greater Pielou’s Evenness score than Cluster 3 (p = 0.0007). Evenness did not differ between Clusters 1 and 2 (p = 0.24). To visualize each case cluster in relation to controls, a PCoA was also generated using Bray–Curtis dissimilarity (Fig. 7). In this analysis, Cluster 2 resistomes were more similar to controls, whereas Cluster 1 resistomes separated along the first and second coordinate with Cluster 3 oriented in between. The difference between the centroids of each case cluster was significant (PERMANOVA p = 0.000999; F = 8.7401).

### Case epidemiological data is linked to specific resistome profiles

Among 25 of the 26 cases with data available, those residing in urban versus rural settings were significantly more likely to have resistome profiles belonging to Clusters 1 or 3 than Cluster 2 (Fisher’s Exact test p = 0.0007). While no significant differences were observed for any of the symptoms across the three clusters, eight of 10 (80.0%) cases reporting bloody stool and five of six (83.3%) cases requiring hospitalization had resistome profiles belonging to Clusters 1 or 3. In addition, 12 of the 17 (70.6%) cases with Cluster 1 or Cluster 3 profiles reported animal contact within one week of illness.

To further explore associations between ARGs, case clusters and epidemiological data, MaAsLin2 was used with cluster and residence type as fixed effects and age and sex as random effects (Table S4). Relative to Cluster 1, significant associations were identified between Cluster 2 profiles and MLS and tetracycline ARGs (C = 5.276026, 2.692487; adjusted p-value = 0.03564, 0.048547, respectively), whereas fosfomycin (C = 3.426063; adjusted p-value = 8.61E−05), aminocoumarin (C = 1.481023; adjusted p-value = 0.001451), and elfamycin (C = 1.303181; adjusted p-value = 0.000107) ARGs were associated with Cluster 3. By contrast, Cluster 1 communities were associated with aminoglycoside (C = − 1.66939; adjusted p-value = 1.30E−09), cationic antimicrobial peptides (C = − 5.03738; adjusted p-value = 1.60E−17) and MDR (C = − 0.47969; adjusted p-value = 0.002346) classes relative to both Clusters 2 and 3. Similar results were determined when using an alternative method, LeFSe, for identifying differentially abundant ARGs among clusters at the class level (Fig. S6).

Intriguingly, trimethoprim was the only ARG class associated with rural residence, while the group level analysis identified *dfhR*, which is important for trimethoprim resistance, to be more common in rural cases (Table S5). Five additional ARGs including *tetA* and *tetB* (tetracycline), *mphA* (macrolides), *aac3* (aminoglycoside)*,* and *ANT3-DPRIME* (aminoglycoside), were also significantly more common in rural versus urban residents.

### Family relation is less influential than health status in shaping the gut resistome during enteric infection

**Figure 8:**
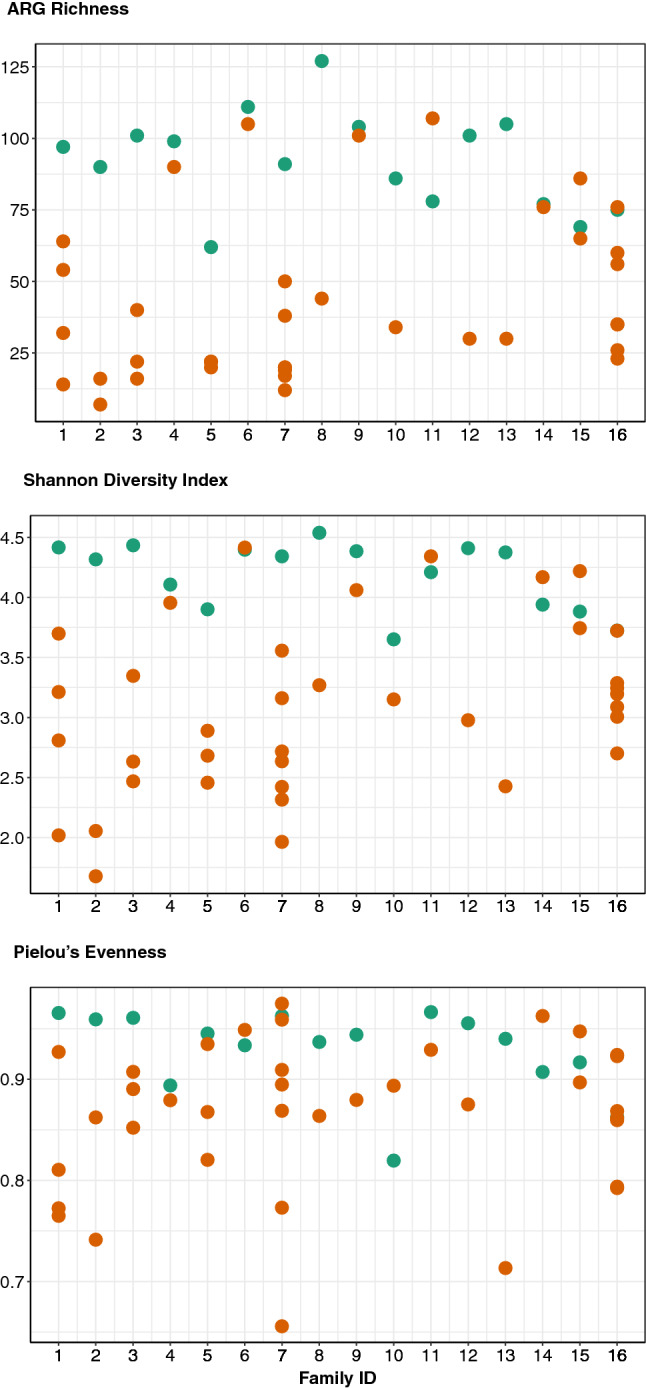
The alpha diversity measures of ARG richness, evenness, and Shannon diversity did not significantly differ by family. However, differing levels of variance were observed among families, particularly when comparing families with one control sample vs. many. Each column in the dotplot represents a single family with one case sample (green circle) and one or more control samples (orange circles). P-values were calculated using the Wilcoxon rank-sum test but none were significant.

**Figure 9:**
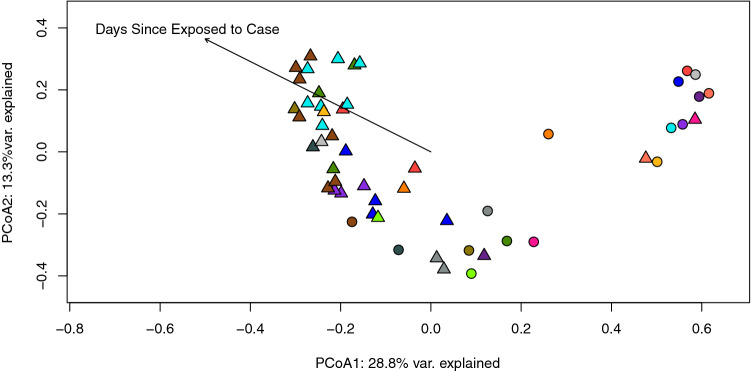
Beta diversity analyses do not reveal clear similarities among 16 separate families as seen in a Principal coordinates analysis (PCoA) plot generated using Bray–Curtis dissimilarity of all samples. Case samples are depicted as circles and controls as triangles; members of the same family are represented by the same color. Environmental factors with potential to influence this ordination were also examined. The variable exploring the number of days since exposure to the case was significantly correlated with the observed ordination. These data were fitted to the ordination using the ‘envfit’ function in R and displayed with a labeled arrow in the plot. The number of days since exposure to an ill family member is correlated to more “normal” looking controls (i.e., controls that are most dissimilar from their corresponding infected cases).

An analysis of 16 families was pursued by comparing case samples to 1–7 family members (controls) who submitted stools 5–21 weeks following the case’s infection. Although no significant differences were observed for richness, evenness, and Shannon diversity by family (Fig. 8), the resistome composition varied considerably. The latter result is supported by an examination of beta diversity metrics since a PCoA revealed little separation among the families with health status contributing to most of the separation (Fig. 9). Some cases and controls within a family, however, were in closer proximity than expected, which is in line with the ARG distribution and abundances by family. With the exception of a few families, resistome composition among cases was clearly distinct from those observed among their related controls (Fig. S7).

To explain the variance observed among families, environmental factors and vectors were fit onto the ordination. These variables included gender, age in years, residence type, and number of days since exposure to the infected family member (controls only). There was a significant correlation between the number of days since exposure and the ordination values (p = 0.001, R2 = 0.543). Interestingly, the directionality of this influence corresponds with controls that were less similar than their associated cases. In other words, the longer the time between a case being infected and a control submitting a stool sample, the less similar the control resistome appeared to its corresponding case. A significant correlation was also observed between residence type and the ordination values (p = 0.019, R2 = 0.113).

Next, we used MaAsLin2 to model family (fixed effect) with health status, residence type, sex, and age as random effects to identify ARG classes and groups associated with specific households while controlling for demographic factors. At the class level, fosfomycin ARGs were significantly associated with family #14 (C = 4.2; adjusted-p = 1.1E−07), while the group level analysis identified three ARGs to be associated with four different families. *fosA* and *acrB*, for example, were significantly associated with family #14 (C = 4.3, 2.0; adjusted-p = 1.1E−06, 9.2E-05, respectively), whereas *acrB* was associated with family #15 (C = 1.3; adjusted-p = 0.007) and *mel* was linked to families #4 and #8 (C = 5.6, 4.4; adjusted-p = 1.8E−06, 5.7E−05, respectively).

## Discussion

Gastrointestinal dysbiosis has been shown to influence and be influenced by the gut microbiota^3,21^. Disease state as it pertains to dysbiosis not only impacts microbial taxa in the gut but can influence the functional composition of this environment as well^22^. Herein, we found that gut communities characterized from the stools of patients with *Campylobacter* infections (cases) had increased resistome diversity relative to healthy family members (controls). The differences observed between cases and controls in this metagenome analysis are consistent with our prior study that used 16S rRNA sequencing to demonstrate discrepancies in microbiota diversity between study groups^3^. It is probable that fluctuations observed in the microbiome and resistome following enteric infection are linked, as changes in microbial composition will inherently shift the relative presence/absence of associated genes. Therefore, the role of enteric infection in driving these fluctuations is of great interest.

The identification of multiple differentially abundant ARG classes and groups in case samples suggests that *Campylobacter* infection influences the composition and diversity of the resistome. Most notable is the relative increase in MDR and fluoroquinolone resistance genes in case samples. *Campylobacter* strains can often harbor these genes^23^, highlighting the possibility that pathogens can transport them into the gut community. Our taxa analysis of case samples, however, estimated the relative abundance of the *Campylobacter* genus to be low (0.28%), while genera such as *Escherichia* were much more abundant. Interestingly, however, many of the MDR ARGs detected among cases were correlated with other taxa such as *Shigella;* for example, *Shigella* was highly correlated with the MDR gene *mdtC*. Multidrug efflux is a common resistance mechanism and therefore carriage of *mdtC* by *Shigella* spp*.* is consistent with previous findings^24^. Nonetheless, detection of *Shigella* raises questions regarding co-infections, which require confirmation via culture or other diagnostic tests. It is also important to note that we did not directly explore genetic architecture, a technique which would more clearly elucidate which microbes harbor specific ARGs of interest. Despite providing preliminary information regarding ARG-harboring taxa, this method assumes that ARGs and taxa co-occurring in similar abundances indicate a correlation, potentially leading to inaccurate associations. Indeed, an association between ARGs and their microbial hosts was observed in a prior study using methods that measured both ARGs and taxa abundance^7^. While fluctuations in the abundance of specific taxa during infection likely change the abundance of ARGs harbored by these taxa, future work employing a more rigorous test assessing key taxa-associated ARGs is needed. It will also be important to examine these communities in a phylogenetic context using tools like UniFrac^25^ when they become more readily available for use with metagenomics data.

It is notable that cases with *Campylobacter* infections had three distinct clusters with differentially abundant ARG profiles. Resistomes belonging to Cluster 2 were more similar to control resistomes, a finding that could point to less perturbed gut communities with a greater initial resilience or to partially recovered communities at the time of sampling. Cluster 1 and Cluster 3 resistomes, however, were either more perturbed by infection or were distinct at the start of the infection. Indeed, it is possible that the trajectory of an individual’s resistome during infection is contingent upon the microbiome composition before infection. Support for this possibility comes from a prior study of *Campylobacter* patients, who had significantly lower taxonomic diversity in their gut communities before infection^26^, an outcome that could be related to varying levels of microbiome resilience^27^. Indeed, studies in mice with varying degrees of microbial imbalance prior to infection demonstrated that disturbed gut communities were more susceptible to infection by *Salmonella enterica* serovar Typhimurium^28^. Another study in chickens observed *Campylobacter* invasion of the cecal microbiome only after substantial changes to the metabolic profile were detected^29^. Direct interactions between the normal gut microbiota and invading pathogens via resource competition, metabolite production, and direct antagonism, coupled with the complexity of pathogen-induced inflammation, have also been shown to influence enteric infections^30,31^. Variable perturbations among individuals is consistent with prior studies showing distinct shifts in the gut microbiota and resistome following antibiotic treatment^32,33^.

Because we could not evaluate patient communities prior to infection onset, an approach that would require a costly and lengthy longitudinal study of healthy individuals, we cannot rule out the possibility that the sampled communities were already distinct. We have utilized a single sample taken during infection (cases) and during a self-described “healthy” period (controls) and therefore, an assessment of microbiome changes over time could not be performed. Longitudinal studies are needed to define the trajectory of microbiome fluctuations in the gut and determine how *Campylobacter* impacts these alterations. Another limitation is the assumption that stool is representative of the human gut microbiome. Previous work has shown that microbial signatures in the stool differ from other gut-related samples from the same individual^34,35^. Since the abundance of different bacterial populations differs in stool, our findings likely represent an underestimate of the actual abundance of taxa and ARGs within the gut. Future studies should therefore examine additional sample types, such as mucosal tissues, to better define how the gut microbiome is impacted by *Campylobacter*.

Specific factors responsible for observed differences between resistome profiles of cases remain elusive. It is possible, however, that geographic location as well as variable exposures and host responses play a role. For example, a prior study reported differences in ARG composition and abundance across land-use sites (rural, urban, and industrial), with ARGs fluctuating seasonally and in accordance with a relevant mobile genetic element (MGE), *int*1^36^. Another study suggested that local anthropic activities, regardless of rural or urban identity, play a role in determining ARG profiles^37^. These findings are consistent with our observation that urban residents were significantly more likely to have a resistome profile belonging to Clusters 1 or 3 than Cluster 2, indicating that unique factors may be important for the expansion of specific ARGs during acute infection. Variation in host immune responses could offer another potential explanation for the observed differences among cases. *Campylobacter* infection elicits activation of NFΚ-B and the production of pro-inflammatory cytokines such as interleukin (IL)-8^38^, though cytokine responses can vary among strains^39^. This variation may contribute to dissimilar levels of inflammation that differentially influence the resident gut microbiota and, at times, benefit the invading pathogen^30,40,41^. Of importance, too, is that specific pathogen features such as the lipooligosaccharide and polysaccharide capsule can impact virulence and host responses^42,43^, while repeated exposure to *Campylobacter* has been linked to local and systemic inflammation in children^44^. Host responses and *Campylobacter* strain characteristics were not evaluated in our study and, hence, we cannot rule out the possibility that some of these factors impacted the resistome profiles observed. Future studies should therefore utilize a larger sample size to further clarify factors that contribute to more perturbed or variable resistomes.

Resistome profiles detected in control samples also provide important information about background levels of resistance due to the presence of specific ARGs and ARG classes. The finding that tetracycline, MLS, and beta-lactam ARGs were more abundant in controls is consistent with two global studies, though gene prevalence was somewhat impacted by geography^9,45^. In the United States, healthy individuals harbored MLS and beta-lactam resistance genes^46^. While the reasons behind the increased abundance of these ARGs in healthy individuals is not clear, it is possible that historic circulation of these drugs in agriculture as well as veterinary and human health has had a long-term impact on the gut microbiome. Comparatively, historical use of tetracyclines in the medical field could also have long-term effects on gut microbes, which has been shown for group B *Streptococcus*^47^, an opportunistic pathogen that commonly resides in the gut. Because ARGs can be horizontally transferred to commensal gut bacteria and are stably maintained in this environment regardless of recent exposure or antibiotic use^48^, detection in healthy control stools could be attributed to prior exposure to antibiotics or acquisition of antibiotic resistant bacteria. This suggestion is consistent with ARGs identified in other cohorts^9,45,46^ and strengthens our assumption that uninfected control samples can be used as a baseline for comparison when analyzing resistomes following pathogen infection.

It is important to note that directly comparing case and control samples presented some challenges. First, control samples were obtained weeks after the related patients had recovered, which prevented an assessment of other factors that may be linked to resistome differences. Indeed, some of the observed variation between cases and controls could be due to factors such as diet and exercise level, which were not measured in this study but have been shown to influence gut communities. Secondly, the sample size of our cases and controls differed. Multiple controls were associated with a single case in some circumstances, while other cases lacked corresponding controls. Regardless of these limitations, however, we were provided with a novel opportunity to conduct a family-based analysis to explore how familial relations may influence the resistome.

Among all 16 families examined, family relation did not appear to outweigh the effect of disease state on the resistome as most cases had different profiles than the controls within each family. Variation in ARG distribution and abundance, however, was observed across families with four families having specific ARGs that were more likely to be shared among their family members. The mismatched number of controls per case, however, makes interpreting these data difficult, as more controls per case may have overestimated the importance of some ARGs. Nonetheless, the analysis exploring environmental associations was notable. Specifically, time since exposure to the infected family member significantly influenced control resistomes; the longer the time period between a case being infected and a control submitting a stool sample, the less similar the resistomes are. Intuitively, this is expected since the longer the period following exposure to a case, the less likely a healthy family member will show signatures of potential infection/crossover.

The level of social closeness among family members may have also played a role in the similarity of their resistomes. A prior study, for instance, noted that the closer the social interaction between two family members (such as between married partners), the more similar their gut microbiome compositions were^49^. Unfortunately, we did not consistently receive information about the relational status of each control, and hence, conclusions about these relationships could not be made. In addition, due to the differing number of household members available per family as well as our hesitancy to exclude samples on a nonrandom basis, the uneven distribution of controls:cases limits our interpretation of these data. Regardless, the provision of multiple control samples enabled us to observe similarities/differences between healthy members of a family in relation to each other and their infected relative, a tenet of this study that may prove useful in future analyses when considering how pathogens impact the gut microbiome.

Collectively, these data demonstrate that patients with *Campylobacter* infections have key differences in the human gut resistome relative to healthy, uninfected individuals. Of great interest, we observed an increase in specific taxa, the diversity of ARGs, and ARGs related to MDR in the patients. These findings substantiate the need for further characterizing the microbiome and resistome in response to perturbations such as those caused by enteric pathogens. Future work should also involve examining bacterial genes found to be differentially abundant between groups or that possessed SNPs within genes linked to antibiotic resistance previously. Indeed, it is likely that periods of flux not only influence the composition of the microbiome, but also its capacity for horizontal gene transfer, which can play a role in the persistence and transmissibility of ARGs and emergence of resistant pathogens.

## Methods

### Study population

Between 2011 and 2015, 26 stools were obtained from patients with *Campylobacter* infections prior to treatment. Most infections were caused by *C. jejuni*, although one patient had *C. coli* and three isolates could not be classified. Samples were collected via the Michigan Department of Health and Human Services (MDHHS) as described^3^. Briefly, stools were added to Cary-Blair transport media, cultured for *Campylobacter* spp., and transported to Michigan State University (MSU). Upon receipt at MSU, stool samples were homogenized, centrifuged and aliquoted for analysis and/or storage at − 80 °C. Metagenomic stool DNA was extracted using the QIAamp DNA Stool Mini Kit (QIAGEN; Valencia, CA) as described^50^. Epidemiological data about demographics, exposures, hospitalization, and symptoms were extracted from the Michigan Disease Surveillance System (MDSS) and household members were contacted for inclusion as study controls. Forty-four healthy household family members submitted a stool 5–21 weeks after the cases’ infection and completed a questionnaire about exposures and symptoms. Sixteen families were included. While 10 cases and 7 controls were not matched to a shared household, they were included in the overall comparative case versus control analyses. County of residence was classified as ‘rural’ or ‘urban’ based on the classification scheme developed by the National Center for Health Statistics^51^ (NCHS). These classifications utilized census data from 2010 while considering the 2013 county designation assigned by the Office of Management and Budget as metropolitan, micropolitan, or noncore, as well as the specific population sizes and city location for metropolitan areas.

Study protocols and consent procedures were performed as described^3^ in accordance with the relevant guidelines and regulations. Approval to conduct the study was granted by the Institutional Review Boards at MSU (IRB #10-736SM), the MDHHS (842-PHALAB), and four hospital laboratories. Each participant and/or their legal guardian was required to provide informed consent prior to enrollment and was given a monetary incentive after each sample was submitted. Data were stripped of all personal identifiable information prior to use.

### Sample preparation and sequencing analysis

Metagenomic DNA from the 70 stools was extracted, sheared, and normalized as described^3^. Library construction was completed using a TruSeq Nano library kit (Illumina, Inc., San Diego, CA, USA) and shotgun sequencing was performed in a series of four sequencing runs on an Illumina HiSeq 2500. Reads were demultiplexed at the MSU Research Technology Support Facility (RTSF). Sequencing run was investigated as a potential source of batch effects prior to analysis of the data; considerable overlap was observed across runs (Fig. S8).

AmrPlusPlus v2.0 was used to perform quality control and align and annotate the metagenomic fragments using the MEGARes 1.0 database^52^. This database was chosen for its comprehensive, hand-curated compilation of ARGs and associated annotation structure containing three hierarchical levels that maximizes the number of representative sequences and lacks cycles or statistical dependencies^52^. Trimmomatic^53^ was used to remove adapters and poor-quality reads. Specifically, the reads were trimmed by removing the three leading and trailing nucleotides, followed by trimming of the 5′ end of the sequence until an average Phred score of > 15 was attained in a sliding window of size four. Short sequences < 36 nucleotides were discarded. If reads matched to adapter sequences with less than or equal to two mismatches, then they were eligible for clipping to ensure adapter removal; successful clipping was dependent on a match score of ≥ 30 using a publicly-accessible adapters file provided on GitHub (https://github.com/BioInfoTools/BBMap/blob/master/resources/adapters.fa).

Metagenomic reads were mapped to the human genome (GRCh38_latest_genomic.fna.gz, downloaded December 2020 from RefSeq) using Burrows-Wheeler Aligner (BWA)^54^; SAMTools^55^ and BEDTools^56^ were used to remove these human genomic sequences from each sample. Following trimming, quality filtering, and host genome removal, 176,686,501 of the 217,104,781 raw paired-end reads were used for downstream analyses. The number of paired-end reads used in the analysis did not significantly differ between cases and controls (p = 0.051). The estimated and actual sequencing coverage were determined using Nonpareil^57^; the average coverage was estimated to be 83.0% (Fig. S9). Average Genome Size (AGS) and the number of genome equivalents (GE) within each sample were quantified using MicrobeCensus^58^. Because AGS analyses have been considered a potential source of bias in gene-based metagenomic comparisons^59^, comparing communities across different sample types may be confounded by varying AGS. Additionally, AGS analyses may provide insight into the ecological capacity of samples; those with a larger AGS may represent generalist taxa, while those with a smaller AGS may represent more specialist species^60^. In our study, AGS was higher in cases (4,406,749.57 bp) versus controls (4,004,525.52 bp) (p = 0.02, Wilcoxon rank sum test; Fig. S10). Because no difference in the number of GE was observed between cases (n = 238.1) and controls (n = 273.5) (p = 0.23, Wilcoxon rank sum test), raw ARG abundance counts were normalized across samples using GE metrics.

### Identification of antimicrobial resistance genes (ARGs)

Non-host FASTQ files resulting from human genome removal were aligned to MEGARes 1.0^52^ using BWA^54^ and SAMTools^55^ with default parameters to classify ARGs present in each sample. Reads were deduplicated and annotated using ResistomeAnalyzer with an identity threshold of ≥ 80% to quantify ARG abundance per sample. RarefactionAnalzyer was performed to obtain the data necessary to assess the adequacy of our sequencing depth. SNPs known to be important for antibiotic resistance were also extracted from the metagenomes using the AmrPlusPlus pipeline^52^. These SNPs were analyzed with the Resistance Gene Identifier (RGI) created in conjunction with The Comprehensive Antibiotic Resistance Database (CARD)^61^ to confirm or reject their presence in ARGs within our samples. In this analysis, however, all ARGs were considered, including those without confirmation of SNP presence. These ARGs were included because they were within a single point mutation and remain relevant even if they serve as a resistance precursor. In future studies, a more in-depth analysis including these SNP data may further illuminate differences between study groups.

Output at the gene level included the target gene, its sequence identity, and putative function; however, output at the group level, or the overall gene- or operon-level group for a given sequence was used. The mechanism level, which indicates the biological mechanism of resistance encoded by each sequence, was also provided as well as the class level representing the antibiotic class relevant to each ARG.

### Identification of microbial taxa

FASTQ reads with the human genome removed via AmrPlusPlus v2.0 were taxonomically annotated using the classifier Kaiju^62^. The NCBI BLAST *nr* database including sequences for bacteria, archaea, viruses, fungi, and microbial eukaryotes was used as a reference. The alignment mode used in Kaiju was ‘greedy’, meaning that a maximum of three mismatches were allowed when identifying taxonomic signatures in sequences. A match length cutoff of 11 nucleotides and the default match score of 65 was used when classifying sequences as well. Raw abundances of reads assigned to taxa were normalized by the estimated number of GE calculated by MicrobeCensus^58^.

### Ecological analyses

Resistome composition was determined by investigating the identity and diversity of ARGs across samples at the gene, group, and class levels. The relative abundance of each ARG was determined per sample by dividing the number of GE-normalized reads for a specific ARG gene, group, or class by the total number of GE-normalized reads for that sample. Alpha and beta diversity metrics, including ordination plots (PCoA) based on Bray–Curtis dissimilarity at the gene level, were determined using the vegan package^63^ in R^64^. The Wilcoxon rank-sum test was used to test for statistical significance between case and control samples (alpha diversity), while PERMANOVA and PERMDISP were used to detect differences in the centroid (mean) and dispersion (degree of spread) across case and control groups (beta diversity).

For the family case–control pairs, the ‘envfit’ function of the vegan package was used to fit environmental variables onto the ordination generated via the PCoA. MaAsLin2^19^ was used to generate log-transformed linear models exploring multivariate associations among resistome features and relevant metadata. Default values were used for all significance cutoffs as well as normalization (total sum scaling; TSS), transformation (log transform), and multiple hypothesis testing correction (Benjamini-Hochberg; BH) with a target False Discovery Rate of 0.05.

### Hierarchical clustering and epidemiological analysis

Case clusters were defined based on the Bray–Curtis dissimilarity among cases at the gene and group levels using the ‘ape’ package^65^ in R and were examined using PCoA and plotted using vegan. The Wilcoxon rank-sum test was used to test for statistical significance between case clusters (alpha diversity), while MaAsLin2^19^ was used to identify differentially abundant ARGs at the group and class level across clusters. For epidemiological analyses, Chi-square tests were used to detect significant differences in epidemiological variables (e.g., patient sex, age, residence (rural vs. urban), and symptoms) between cases and controls and identify associations with case clusters.

### Ethical approval

Study protocols and consent procedures were performed in accordance with the relevant guidelines and regulations set by the Declaration of Helsinki. Informed consent was obtained from all participants and/or their legal guardians prior to enrollment and data were stripped of personal identifying information. Final approval to conduct the study was granted by the Institutional Review Boards at MSU (IRB #10-736SM), the MDHHS (842-PHALAB), and the four participating hospital laboratories as described previously^3^.

## Supplementary Information

Supplementary Information.

## Data Availability

The paired-end metagenome raw reads analyzed in the current study are available in the NCBI repository under BioProject PRJNA660443 (BioSamples SAMN15958881 to SAMN15958950). Metadata associated with the samples discussed in this study are also available within the BioProject. Bioinformatic scripts can be found on GitHub at https://github.com/ZoeHansen/PAPER_Hansen_ScientificReports_2021.
