# Supplementary material for: Comparing gut resistome composition among patients with acute Campylobacter infections and healthy family members
Source: Sci Rep. 2021 Nov 16;11:22368. doi: 10.1038/s41598-021-01927-7 (PMC8595376; doi:10.1038/s41598-021-01927-7)

**Comparing gut resistome composition among patients with acute *Campylobacter* infections and healthy family members**

Zoe A. Hansen[1,3], Wonhee Cha[1], Brian Nohomovich[1], Duane W. Newton[4], Paul Lephart[4], Hossein Salimnia[5], Walid Khalife[6], Ashley Shade[1,2,3], James T. Rudrik[7], and Shannon D. Manning[1,3*]

*[1]Departments of Microbiology and Molecular genetics and [2]Plant, Soil and Microbial Sciences, and the [3]Ecology, Evolution, and Behavior Program, Michigan State University, East Lansing, MI, 48824, USA; [4]University of Michigan, Ann Arbor, MI, 48109, [5]Wayne State University, Detroit, MI 48202, [6]Sparrow Hospital, Lansing, MI 48912; and [7]Michigan Department of Health and Human Services, Bureau of Laboratories, Lansing, MI 48913*

**<u>Email addresses</u>**

Zoe A. Hansen: hansenzo@msu.edu; Wonhee Cha: wonhee.cha@sva.se; Brian Nohomovich: nohomovi@msu.edu; Duane Newton: dnewton@navidx.com; Paul Lephart: plephart@med.umich.edu; Hossein Salimnia: HSalimni@dmc.org; Walid Khalife: Walid.Khalife@Sparrow.Org; Ashley Shade: shadeash@msu.edu; James Rudrik: RudrikJ@michigan.gov; and *Shannon D. Manning: mannin71@msu.edu (Corresponding author)

**Table S1. Characteristics of 26 patients with *Campylobacter* infections (cases) and 44 healthy individuals (controls).**

| Characteristic | Cases No (%) | Controls No (%) | *p*-value‡ |
|---|---|---|---|
| **Demographics** | | | |
| Age | | | 0.093 |
| 0-9 years | 8 (30.7) | 17 (38.7) | |
| 10-18 years | 0 (0.0) | 4 (9.1) | |
| 19-64 years | 13 (50.1) | 21 (47.7) | |
| ≥65 years | 5 (19.2) | 2 (4.5) | |
| Sex | | | 0.083 |
| Male | 9 (34.6) | 26 (59.1) | |
| Female | 17 (65.4) | 18 (40.9) | |
| Residence | | | 0.378 |
| Rural | 11 (44.0) | 11 (29.7) | |
| Urban | 14 (56.0) | 26 (70.3) | |

Note: Not all variables in each column added up to the total number of individuals because of missing data for some variables.

‡ p-values were calculated using the Chi-Square test or Fisher's exact test for variables with n≤ 5 in at least one cell.

**Table S2**. **Differentially abundant antimicrobial resistance genes (ARGs) detected in stool samples from cases and controls.**

| Group (gene) | Association | Coefficient | Standard Error | p-value | Adjusted p-value | ARG class |
|---|---|---|---|---|---|---|
| *cpxAR* | Controls | -4.2583446 | 0.513936708 | 1.01E-11 | 5.17E-11 | Multidrug resistance |
| *mdtC* | Controls | -3.8261348 | 0.439477581 | 1.15E-12 | 9.08E-12 | Multidrug resistance |
| *parE* | Controls | -3.1825883 | 0.361985735 | 8.02E-13 | 6.98E-12 | Fluoroquinolone resistance |
| *parC* | Controls | -3.014637 | 0.248540229 | 1.21E-18 | 1.06E-16 | Fluoroquinolone resistance |
| *tetQ* | Controls | 3.02600618 | 0.490406412 | 4.27E-08 | 8.65E-08 | Tetracycline resistance |
| *cfx* | Controls | 3.72226841 | 1.012164326 | 0.000466 | 0.000654 | Class A beta-lactamase |
| *cbla* | Controls | 4.17780963 | 0.686690753 | 6.06E-08 | 1.15E-07 | Class A beta-lactamase |
| *tetW* | Controls | 4.77784686 | 0.754448744 | 2.21E-08 | 5.49E-08 | Tetracycline resistance |

Gene groups identified with a coefficient ≥|3.0| using MaAsLin2 (Mallick et al. *bioRxiv* 2021, doi:10.1101/2021.01.20.427420) with health status (case vs. control) included as a fixed effect and residence type, age, and sex as random effects. Genes with negative coefficients are more abundant in cases, while positive coefficients are more abundant in control samples.

**Table S3. Correlation values between highly abundant antimicrobial resistant genes (ARGs) and specific taxa detected in *Campylobacter* cases**.

| ARG | Target taxa | Correlation | P value |
|---|---|---|---|
| *mdtC* | *Shigella* | 0.885812 | 1.79E-09 |
| *parE* | *Pseudoalteromonas* | 0.881176 | 2.82E-09 |
| *gyrA* | *Pseudoalteromonas* | 0.873312 | 5.82E-09 |
| *gyrB* | *Pseudoalteromonas* | 0.870085 | 7.74E-09 |
| *gyrA* | *Trabulsiella* | 0.851064 | 3.6E-08 |
| *parC* | *Pseudoalteromonas* | 0.85094 | 3.63E-08 |
| *cpxAR* | *Pseudoalteromonas* | 0.839316 | 8.4E-08 |
| *parE* | *Siccibacter* | 0.82492 | 2.17E-07 |
| *gyrA* | *Siccibacter* | 0.818727 | 3.19E-07 |
| *pare* | *Trabulsiella* | 0.813223 | 4.43E-07 |
| *gyrB* | *Kosakonia* | 0.812522 | 4.61E-07 |
| *gyrA* | *Phytobacter* | 0.811464 | 4.91E-07 |
| *gyrB* | *Phytobacter* | 0.809267 | 5.57E-07 |
| *parC* | *Siccibacter* | 0.80311 | 7.88E-07 |
| *gyrB* | *Siccibacter* | 0.796919 | 1.1E-06 |
| *rpoB* | *Trabulsiella* | 0.796432 | 1.13E-06 |
| *pare* | *Phytobacter* | 0.796024 | 1.16E-06 |
| *gyrB* | *Trabulsiella* | 0.795543 | 1.19E-06 |
| *cpxAR* | *Phytobacter* | 0.793487 | 1.32E-06 |
| *mdtC* | *Pseudoalteromonas* | 0.789402 | 1.64E-06 |
| *gyrA* | *Pluralibacter* | 0.789195 | 1.66E-06 |
| *gyrA* | *Klebsiella* | 0.787485 | 1.81E-06 |
| *mdtC* | *Rhodococcus* | 0.785091 | 2.04E-06 |
| *cpxAR* | *Siccibacter* | 0.784193 | 2.13E-06 |
| *rpoB* | *Siccibacter* | 0.783009 | 2.26E-06 |
| *parC* | *Phytobacter* | 0.781823 | 2.4E-06 |
| *parC* | *Trabulsiella* | 0.775251 | 3.3E-06 |
| *parE* | *Serratia* | 0.773807 | 3.54E-06 |
| *gyrB* | *Pluralibacter* | 0.766154 | 5.05E-06 |
| *gyrB* | *Pantoea* | 0.76547 | 5.21E-06 |
| *gyrA* | *Serratia* | 0.763207 | 5.77E-06 |
| *parC* | *Pseudescherichia* | 0.762122 | 6.06E-06 |
| *parE* | *Pluralibacter* | 0.761156 | 6.33E-06 |
| *mdtC* | *Phytobacter* | 0.755751 | 8.03E-06 |
| *gyrB* | *Klebsiella* | 0.755214 | 8.22E-06 |
| *gyrA* | *Lelliottia* | 0.755175 | 8.23E-06 |
| *gyrA* | *Yersinia* | 0.752949 | 9.06E-06 |
| *parC* | *Pluralibacter* | 0.751795 | 9.52E-06 |

**Table S4. Differentially abundant antimicrobial resistance gene (ARG) classes detected in 25 cases with specific resistome profiles (clusters) determined by hierarchical clustering.**

| ARG Class | Association | Coefficient | Standard Error | p-value | Adjusted p-value |
|---|---|---|---|---|---|
| MLS | Cluster 2 | 5.276026 | 1.888096 | 0.011031 | 0.03564 |
| Tetracyclines | Cluster 2 | 2.692487 | 1.027064 | 0.016182 | 0.048547 |
| Multidrug resistance | Cluster 2 | -0.45724 | 0.146904 | 0.005269 | 0.018441 |
| CAP | Cluster 2 | -0.48417 | 0.203713 | 0.027054 | 0.066838 |
| Aminoglycosides | Cluster 2 | -0.59723 | 0.172927 | 0.002378 | 0.009989 |
| Fluoroquinolones | Cluster 2 | -0.72234 | 0.206928 | 0.002273 | 0.009989 |
| Aminocoumarins | Cluster 2 | -0.98069 | 0.428388 | 0.032525 | 0.075891 |
| Rifampin | Cluster 2 | -1.03029 | 0.428546 | 0.025522 | 0.066838 |
| Sulfonamides | Cluster 2 | -1.59707 | 0.493373 | 0.004139 | 0.015803 |
| Fosfomycin | Cluster 3 | 3.426063 | 0.691953 | 8.61E-05 | 0.001127 |
| Aminocoumarins | Cluster 3 | 1.481023 | 0.404347 | 0.001451 | 0.009989 |
| Elfamycins | Cluster 3 | 1.303181 | 0.274124 | 0.000107 | 0.001127 |
| Fluoroquinolones | Cluster 3 | 0.682634 | 0.195489 | 0.002225 | 0.009989 |
| Multidrug resistance | Cluster 3 | -0.47969 | 0.13866 | 0.002346 | 0.009989 |
| Bacitracin | Cluster 3 | -0.93482 | 0.389323 | 0.026071 | 0.066838 |
| Aminoglycosides | Cluster 3 | -1.66939 | 0.163222 | 1.30E-09 | 2.74E-08 |
| CAP | Cluster 3 | -5.03738 | 0.19228 | 1.60E-17 | 6.72E-16 |
| Beta-lactams | Urban | 0.460124 | 0.233892 | 0.062505 | 0.135027 |
| Trimethoprim | Urban | -3.5159 | 0.905585 | 0.000912 | 0.007657 |

ARG classes were identified using MaAsLin2 (Mallick et al. *bioRxiv* 2021, doi:10.1101/2021.01.20.427420) with case cluster and residence type as fixed effects and age and sex as random effects. Coefficients for the Cluster association were calculated using Cluster 1 as the reference groups, while the urban association used rural residence as the reference. A negative coefficient for Cluster 3, for instance, indicates that Cluster 1 is positively associated with a given class (e.g., aminoglycosides). Some classes were negatively associated with both Clusters 2 and 3 indicating a positive association with Cluster 1. CAP = cationic antimicrobial peptides

**Table S5**. **Differentially abundant genes detected among cases living in urban versus rural settings.**

| Group (gene) | Association | Coefficient | Standard Error | p-value | Adjusted p-value | ARG class |
|---|---|---|---|---|---|---|
| *pbp4B* | Urban | 1.44670 | 0.632259 | 0.033228 | 0.110403 | Beta-lactam resistance |
| *tetA* | Urban | -0.8197 | 0.29973 | 0.012413 | 0.049812 | Tetracycline resistance |
| *tetB* | Urban | -0.8725 | 0.305991 | 0.010208 | 0.04321 | Tetracycline resistance |
| *mphA* | Urban | -1.20645 | 0.341498 | 0.002378 | 0.013606 | Macrolide resistance |
| *tetR* | Urban | -1.55327 | 0.63068 | 0.02351 | 0.084223 | Tetracycline resistance |
| *dhfR* | Urban | -1.64111 | 0.425635 | 0.000917 | 0.006028 | Trimethoprim resistance |
| *aac3* | Urban | -1.80729 | 0.517081 | 0.002157 | 0.012816 | Aminoglycoside resistance |
| *ANT3-DPRIME* | Urban | -1.95451 | 0.627014 | 0.005213 | 0.025979 | Aminoglycoside resistance |

Gene groups identified using MaAsLin2 (Mallick et al. *bioRxiv* 2021, doi:10.1101/2021.01.20.427420) with Cluster and residence type included as fixed effects and age and sex as random effects. Rural residence is the reference group and hence, genes with negative coefficients are more abundant in rural cases, while positive coefficients are more abundant in urban cases.

**Figure S1. Linear discriminant analysis (LDA) scores showing differentially abundant antimicrobial resistance gene (ARG) classes by health status.** The classes shown registered an LDA score >2.0. The bars shown in orange indicate ARG classes that were more abundant in controls, while green bars show ARG classes that were more abundant in cases. In controls, tetracycline ARGs had the greatest LDA score (5.3; p=8.34e-10) followed by beta-lactam and the Macrolide, Lincosamide, Streptogramin (MLS) ARG classes (LDA=4.6, 4.6; p=0.002, 0.0002, respectively). Ten classes were more abundant in cases, with MDR (LDA=5.2; p=1.68e-09), fluoroquinolones (LDA=4.6; p=8.18e-11), and rifampin ARGs (LDA=4.4; p=3.07e-10) having the highest scores. CAP = cationic antimicrobial peptides; MDR = multidrug resistance.

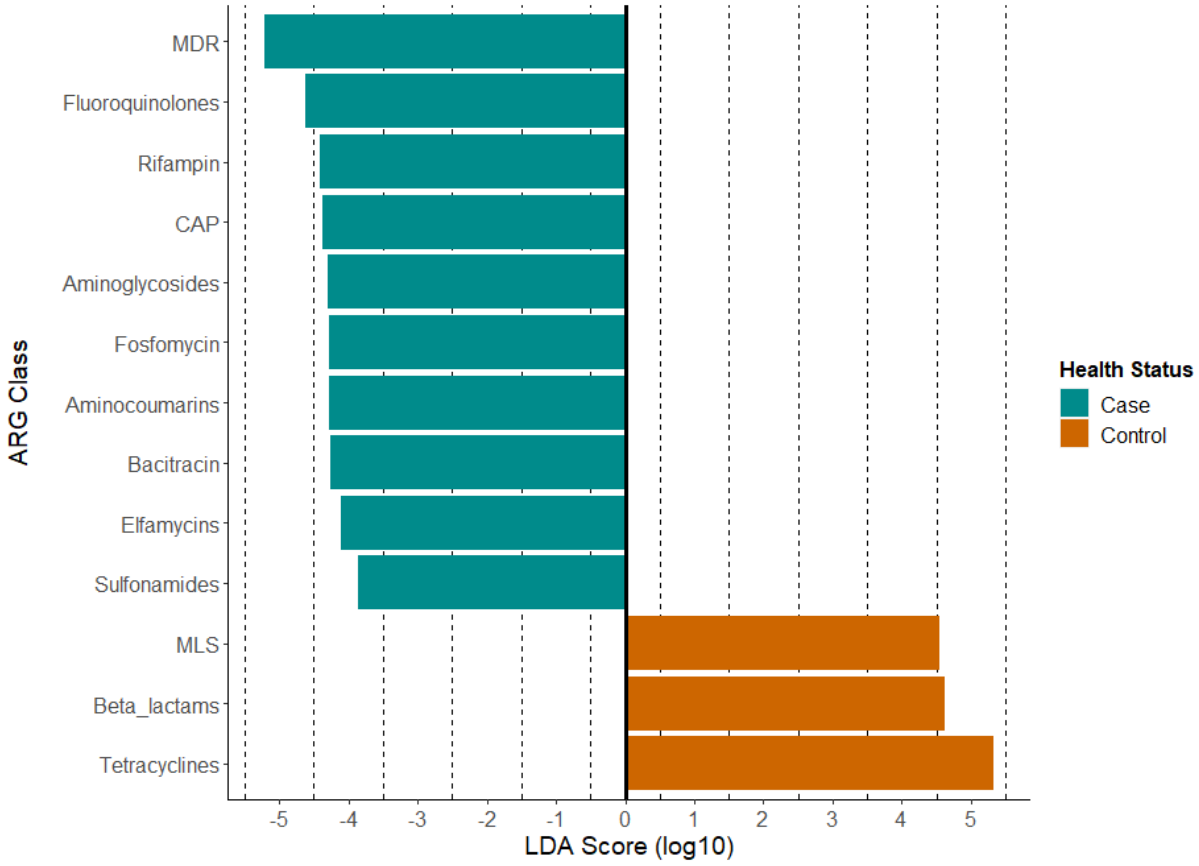

**Figure S2. Linear discriminant analysis (LDA) scores for differentially abundant antimicrobial resistance genes (ARGs) at the group (gene) level by health status.** Each ARG gene included in this plot registered an LDA score >4.0. The orange bars show ARG genes that were more abundant in controls, whereas green bars show genes that were more abundant in cases. In all, 93 of 153 features were differentially abundant between cases and controls. Of these, 12 were more abundant in controls with *tetQ*, *tetW,* and *cfx* predominating, while 81 were more abundant in cases; *rpoB*, *mdtC* and DNA gyrase genes, *gyrB* and *gyrA* predominated in the latter.

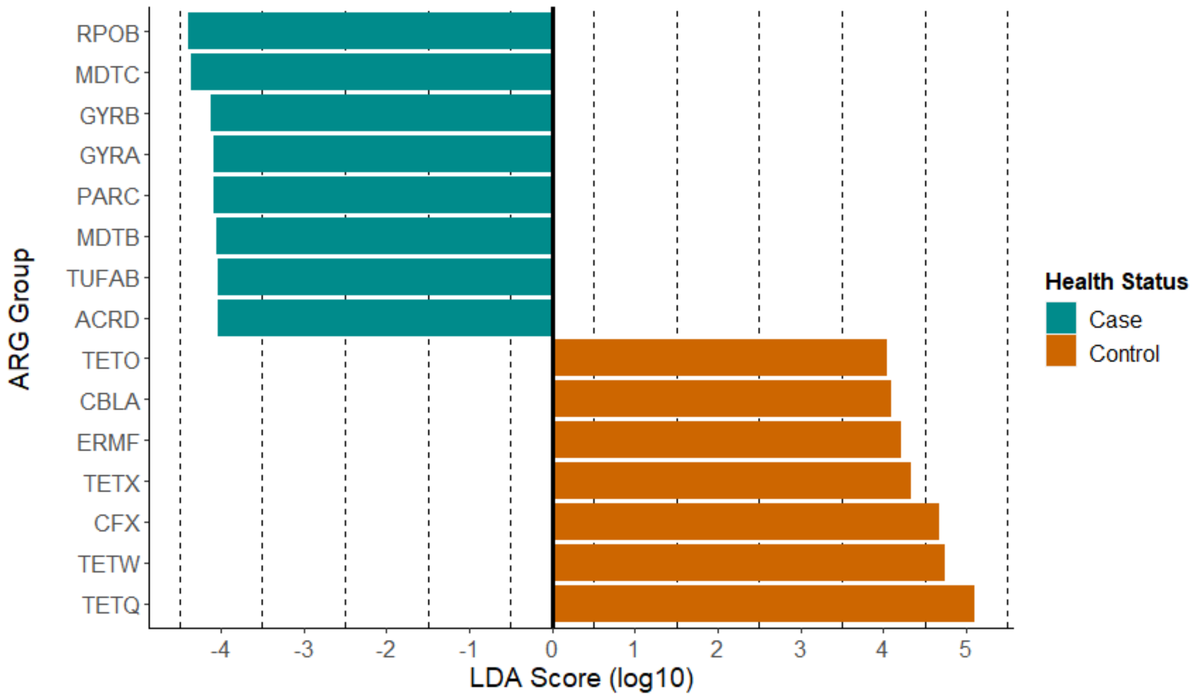

**Figure S3. Controls display higher taxonomic diversity than cases.** Three measures of alpha diversity (Richness, Shannon diversity, and Pielou's Evenness, respectively) are shown for microbial taxonomy among samples. The median of each measure is indicated by the thick black bar in each box and the first and third quartiles are represented by the bottom and top of the box, respectively; jittered points (circles and triangles) show variation within each sample type. Outlying points within each group are indicated by the black dots associated with each boxplot. P-values were calculated using the Wilcoxon rank-sum test and are shown above the comparison bar within each plot.

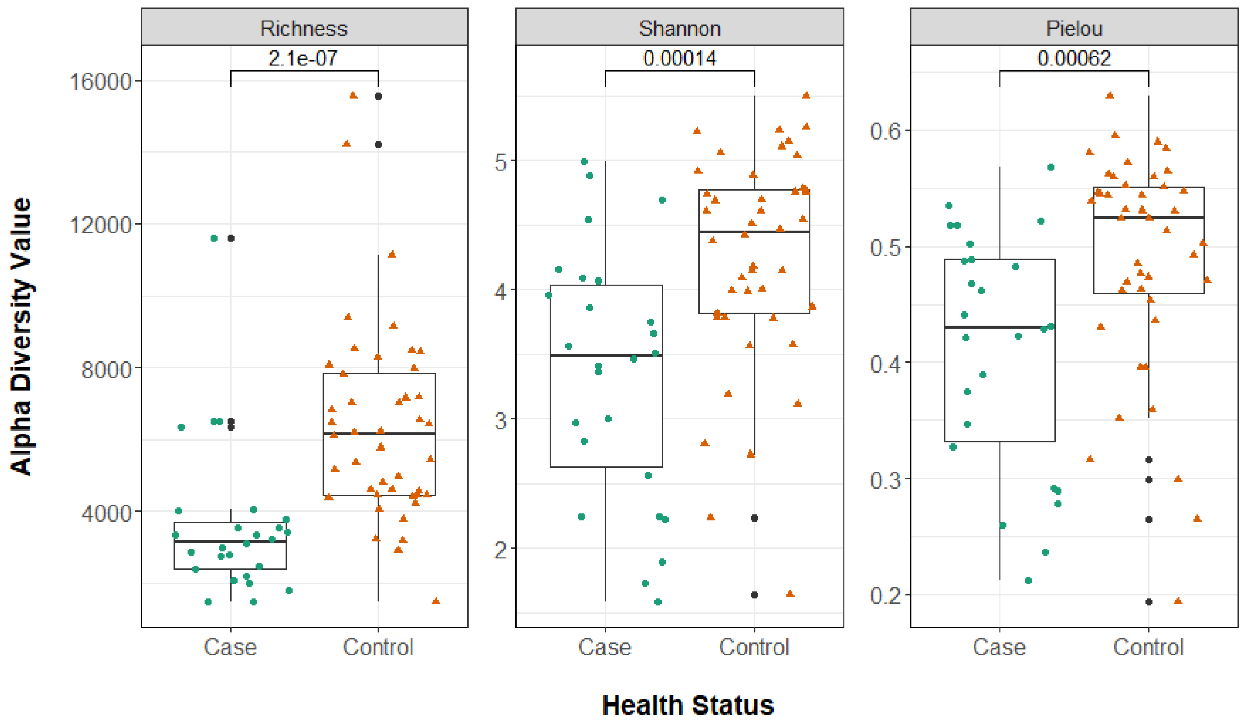

**Figure S4. Actual abundances of bacterial taxa differ considerably between cases and controls.** Rank abundance plots display the average number of reads assigned to bacterial genera and phyla for cases (A, C) and controls (B, D) in decreasing order. The top-10 genera and phyla were determined using the highest average number of reads assigned among cases or controls. All remaining genera or phyla were combined and summed to comprise the group "Other", shown in the plots below.
Note: the y-axis has different scales in each abundance plot.

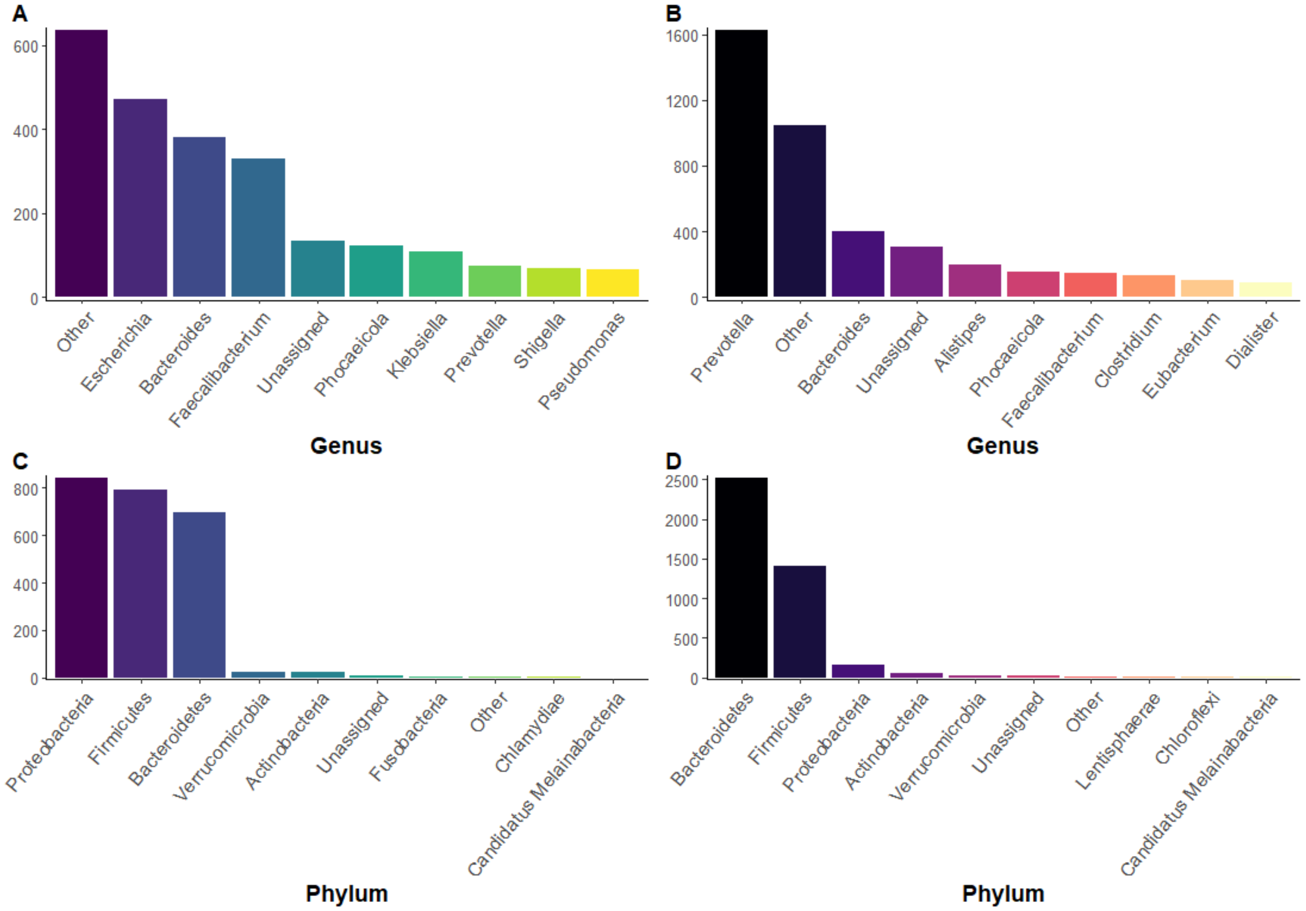

**Figure S5. Hierarchical clustering reveals three distinct resistome profiles among the cases.** Average linkage hierarchical clustering at the gene level was performed based on the Bray-Curtis dissimilarity. Two primary clusters, Cluster 1 and Cluster 2, were identified as well as one outgroup (Cluster 3). Case sample numbers are indicated and colored based on the resistome cluster.

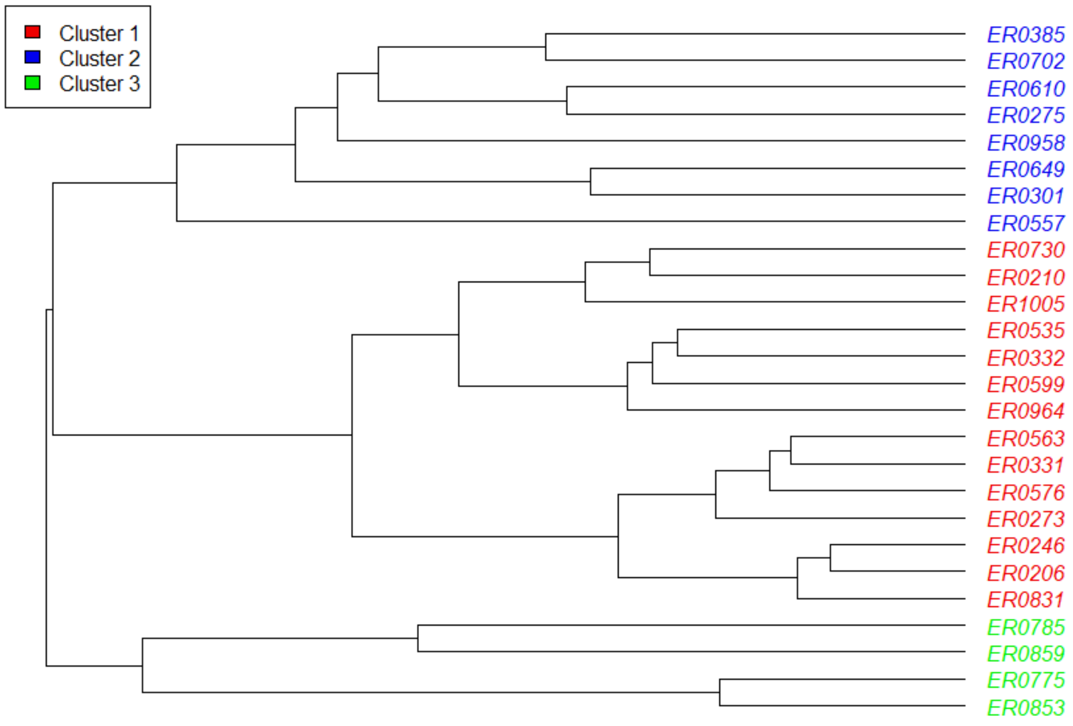

**Figure S6. Linear discriminant analysis (LDA) showing differentially abundant antimicrobial resistance gene (ARG) classes between case clusters.** The classes shown here each registered an LDA score >2.0. The bars shown in red indicate ARG classes that were more abundant in case Cluster 1; blue bars show ARG classes that were more abundant in case Cluster 2; green bars indicate ARG classes more abundant in Cluster 3. MLS = Macrolide, Lincosamide, Streptogramin; CAP = cationic antimicrobial peptides; MDR = multidrug resistance.

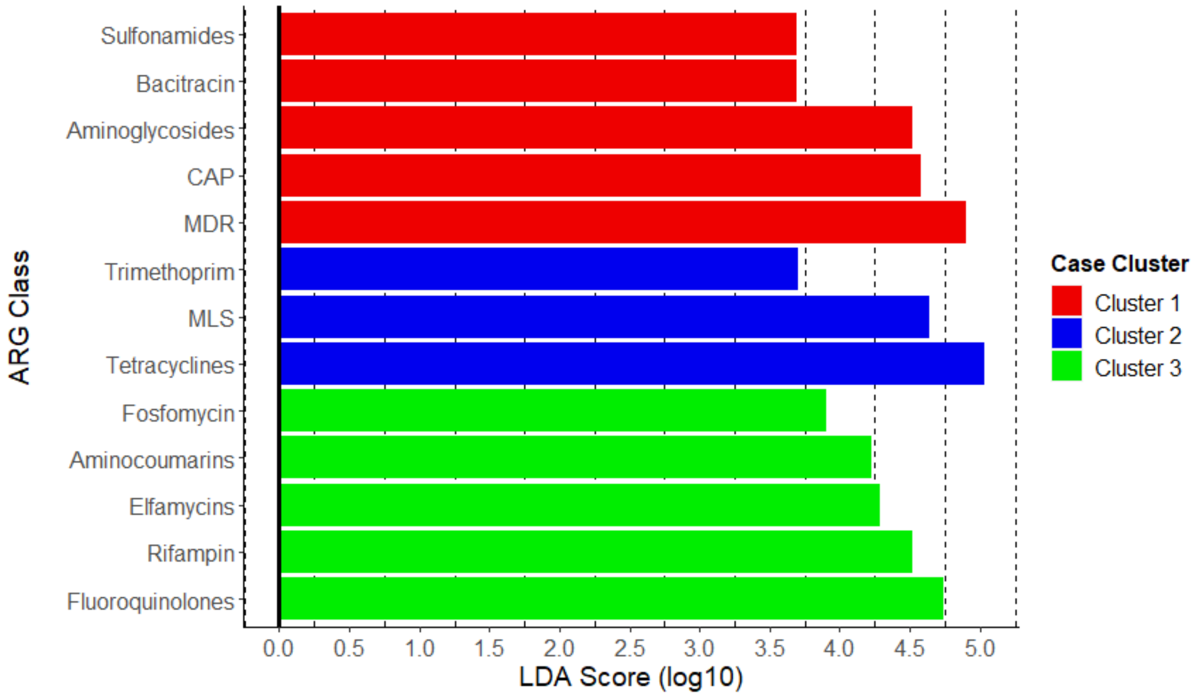

**Figure S7. Relative abundance of ARG classes varies across families but maintains the case versus control dichotomy in most circumstances.** The relative abundance of ARGs assigned to 18 different antibiotic classes is shown with each column representing the resistome from one individual. Each set of numbered plots is faceted by family ID with the left-most column representing the infected individual (cases) in each family; the remaining columns in a family represent 1-7 healthy controls. Relative abundances were determined using raw ARG abundances normalized by the approximate number of genome equivalents in the sample. CAP = cationic antimicrobial peptides; MLS = Macrolide, Lincosamide, Streptogramin; MDR = Multidrug resistance.

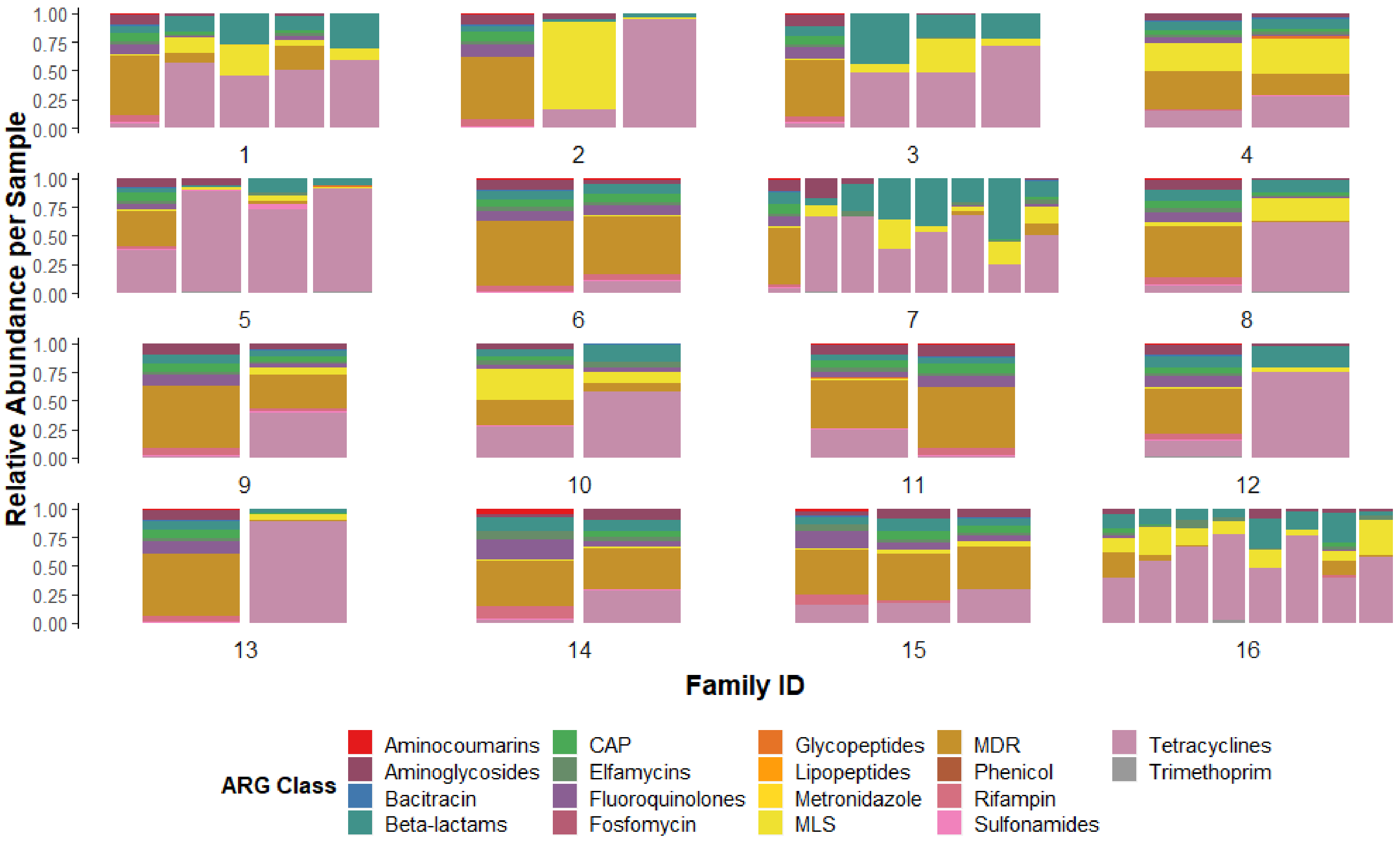

**Figure S8. Sequencing run does not appear to impact resistome similarity among cases and controls.** A Principal Coordinates Analysis (PCoA) plot of case (circles) and control (triangles) resistomes based on Bray-Curtis dissimilarity at the ARG gene level. The first and second coordinate are shown with their respective percentage of explained variance. Sequencing run is denoted by color: Red=Run 1; Blue=Run 2; Green=Run 3; Yellow=Run 4, while patients reporting use of antibiotics are indicated by square data points. Notably, there is considerable overlap among all four sequencing runs. Although a test indicated that the centroids of each run were different (PERMANOVA p=0.000999; F=3.3029) as well as the dispersion of points within each run (PERMDISP p=0.001; F=10.152), this result is attributed to the unequal sample sizes across runs. Run 4, for instance, contains just one sample, whereas Runs 1-3 contain 25, 16, and 28 samples, respectively. Therefore, the difference in centroid and dispersion is expected.

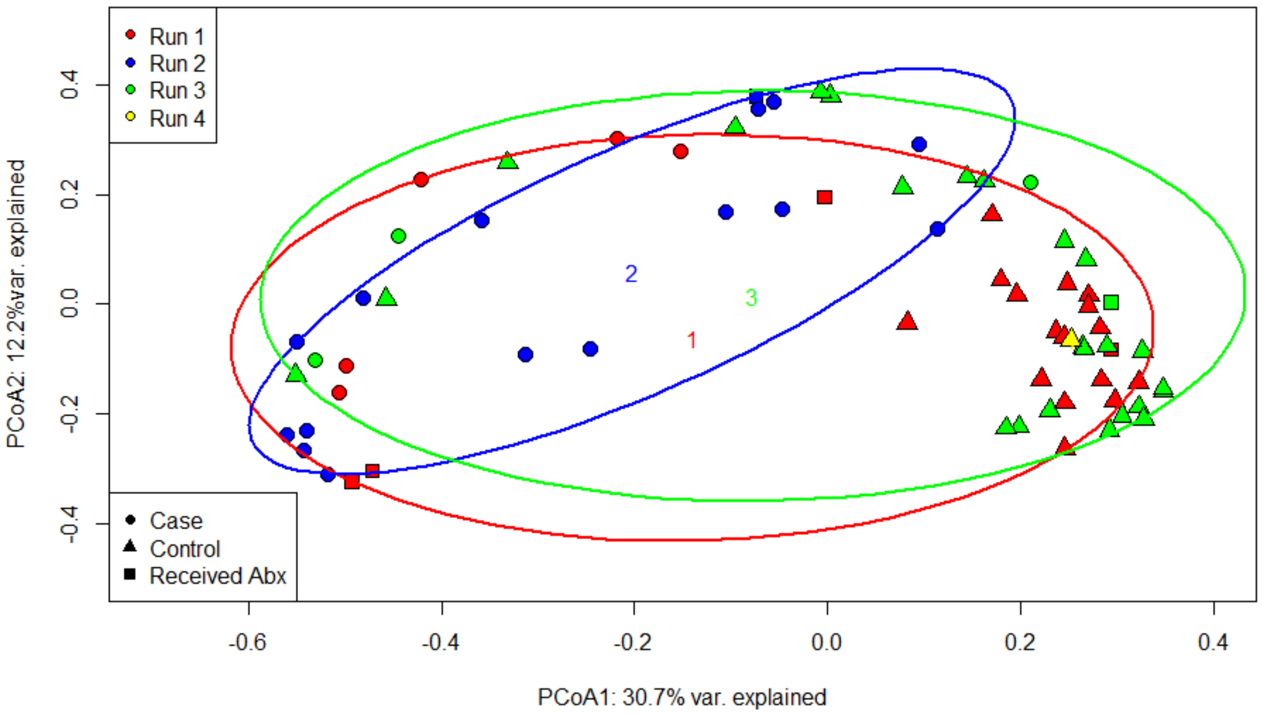

**Figure S9. Estimated sequencing coverage curves for cases and controls.** The estimated coverage (S-curves) and actual coverage (open circles) for case (n=26) and control (n=44) samples evaluated in this study. Each colored S-curve represents a single sample. Arrows at the bottom of the graph represent the Nonpareil index of sequence diversity, which is a measure of community complexity in sequence space; the mean Nonpareil diversity was 17.32 consistent with other stool samples documented with this tool. Dotted red lines represent 100% coverage and 95% coverage, respectively. The overall mean coverage for cases and controls was 83.0%.

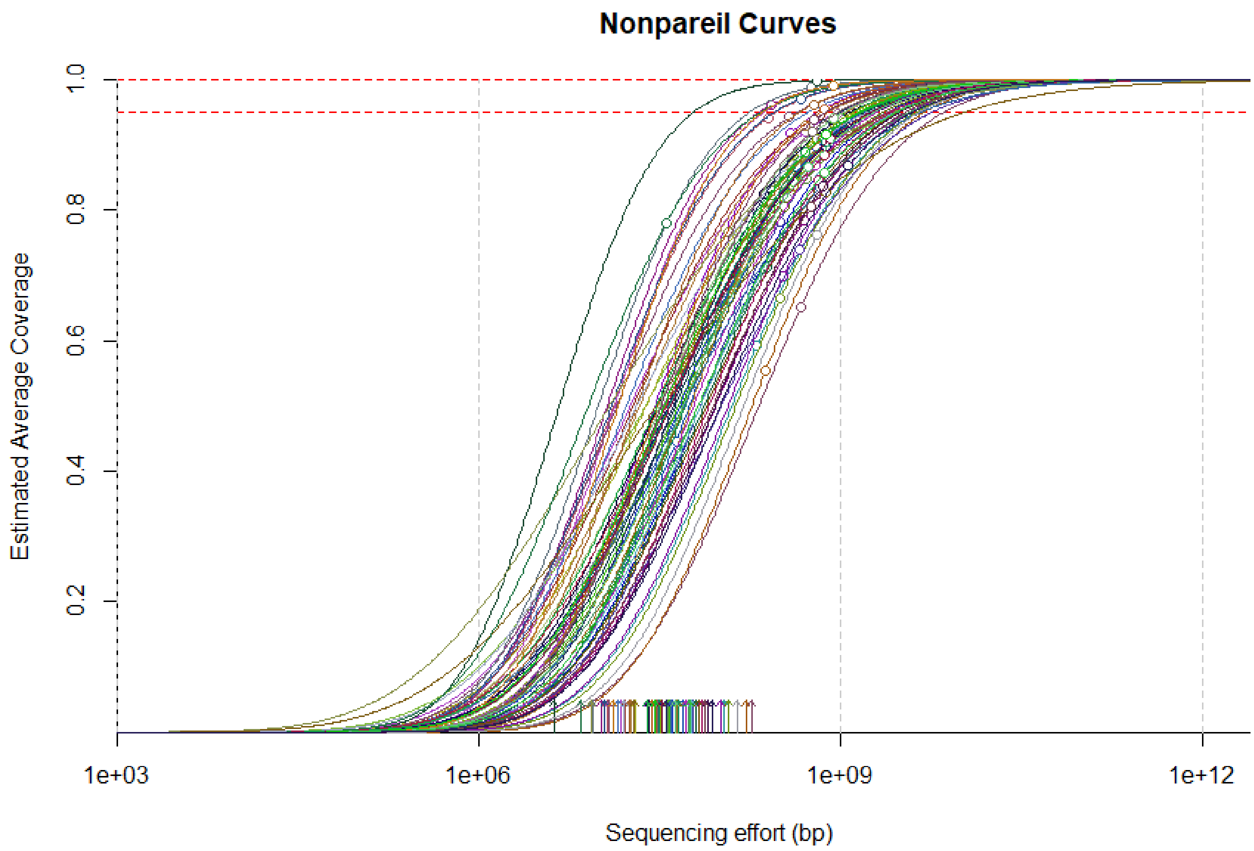

**Figure S10. Comparing the average genome size and number of genome equivalents among case and control samples.** The median of each measure is shown by the black horizontal bar in each box. The first and third quartiles are indicated by the bottom and top of each box, respectively. Points (circles and triangles) are displayed to show variation within the sample types. Outliers within each group are indicated by the black dots.  P-values comparing the difference between cases and controls were calculated using a Wilcoxon rank sum test and are shown above the comparison bar for each metric. Cases = cyan; controls = orange.

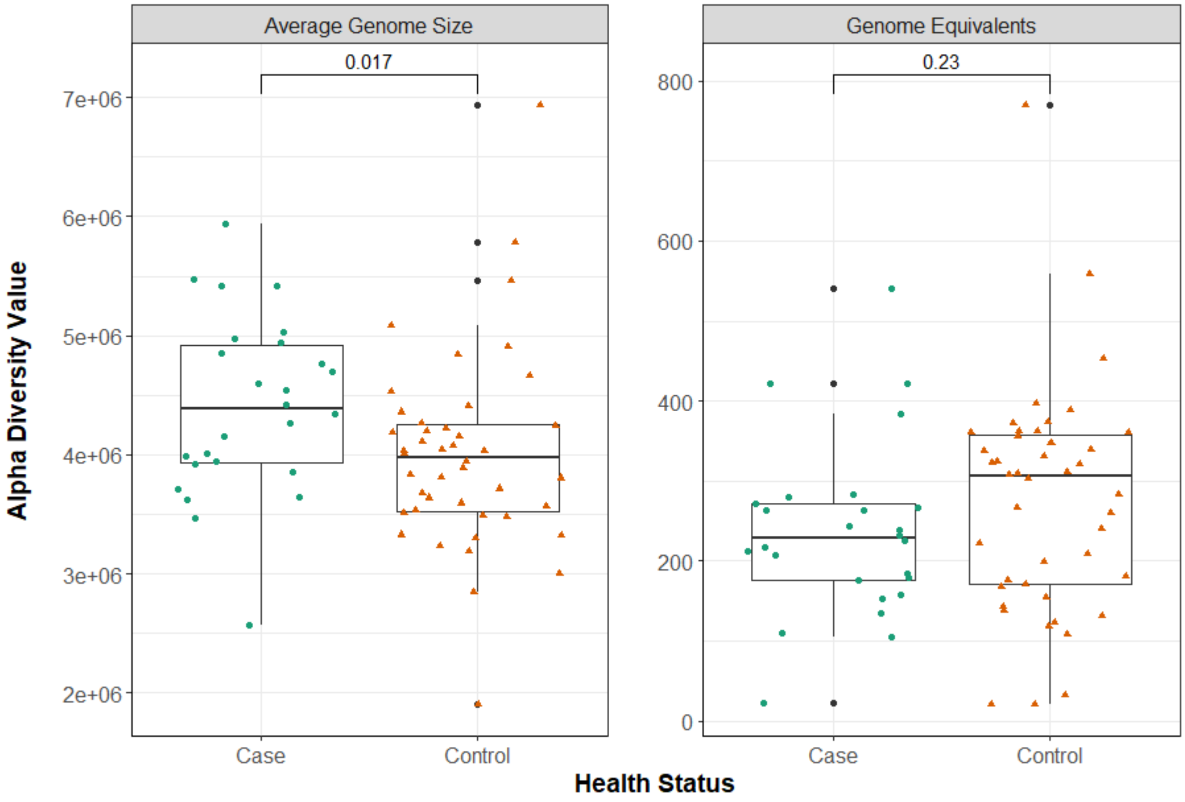

Supplement: Supplementary file 1 — Supplementary Information. [file 41598_2021_1927_MOESM1_ESM.pdf]
